# An Algorithm for Accurate Marker-Based Gait Event Detection in Healthy and Pathological Populations During Complex Motor Tasks

**DOI:** 10.3389/fbioe.2022.868928

**Published:** 2022-06-02

**Authors:** Tecla Bonci, Francesca Salis, Kirsty Scott, Lisa Alcock, Clemens Becker, Stefano Bertuletti, Ellen Buckley, Marco Caruso, Andrea Cereatti, Silvia Del Din, Eran Gazit, Clint Hansen, Jeffrey M. Hausdorff, Walter Maetzler, Luca Palmerini, Lynn Rochester, Lars Schwickert, Basil Sharrack, Ioannis Vogiatzis, Claudia Mazzà

**Affiliations:** ^1^ Department of Mechanical Engineering, Insigno Institute for In Silico Medicine, The University of Sheffield, Sheffield, United Kingdom; ^2^ Department of Biomedical Sciences, University of Sassari, Sassari, Italy; ^3^ Translational and Clinical Research Institute, Faculty of Medical Sciences, Newcastle University, Newcastle Upon Tyne, United Kingdom; ^4^ Department for Geriatric Rehabilitation, Robert-Bosch-Hospital, Stuttgart, Germany; ^5^ Department of Electronics and Telecommunications, Politecnico Di Torino, Torino, Italy; ^6^ Centre for the Study of Movement, Cognition and Mobility, Tel Aviv Sourasky Medical Centre, Tel Aviv, Israel; ^7^ Department of Neurology, University Hospital Schleswig-Holstein, Campus Kiel, Kiel University, Kiel, Germany; ^8^ Department of Physical Therapy, Sackler Faculty of Medicine, Sagol School of Neuroscience, Tel Aviv University, Tel Aviv, Israel; ^9^ Department of Orthopaedic Surgery, Rush Alzheimer’s Disease Center, Rush University Medical Center, Chicago, IL, United States; ^10^ Department of Electrical, Electronic, and Information Engineering “Guglielmo Marconi”, University of Bologna, Bologna, Italy; ^11^ Health Sciences and Technologies–Interdepartmental Center for Industrial Research (CIRI-SDV), University of Bologna, Bologna, Italy; ^12^ The Newcastle Upon Tyne Hospitals NHS Foundation Trust, Newcastle Upon Tyne, United Kingdom; ^13^ Department of Neuroscience, Sheffield NIHR Translational Neuroscience BRC, Sheffield Teaching Hospitals NHS Foundation Trust, Sheffield, United Kingdom; ^14^ Department of Sport, Exercise and Rehabilitation, Northumbria University, Newcastle Upon Tyne, United Kingdom

**Keywords:** gait analysis, spatio-temporal gait parameters, gait cycle, stride length, stride duration, stride speed, stereophotogrammetry

## Abstract

There is growing interest in the quantification of gait as part of complex motor tasks. This requires gait events (GEs) to be detected under conditions different from straight walking. This study aimed to propose and validate a new marker-based GE detection method, which is also suitable for curvilinear walking and step negotiation. The method was first tested against existing algorithms using data from healthy young adults (YA, *n* = 20) and then assessed in data from 10 individuals from the following five cohorts: older adults, chronic obstructive pulmonary disease, multiple sclerosis, Parkinson’s disease, and proximal femur fracture. The propagation of the errors associated with GE detection on the calculation of stride length, duration, speed, and stance/swing durations was investigated. All participants performed a variety of motor tasks including curvilinear walking and step negotiation, while reference GEs were identified using a validated methodology exploiting pressure insole signals. Sensitivity, positive predictive values (PPV), F1-score, bias, precision, and accuracy were calculated. Absolute agreement [intraclass correlation coefficient ( $ICC_{2,1}$ )] between marker-based and pressure insole stride parameters was also tested. In the YA cohort, the proposed method outperformed the existing ones, with sensitivity, PPV, and F1 scores ≥ 99% for both GEs and conditions, with a virtually null bias (<10 ms). Overall, temporal inaccuracies minimally impacted stride duration, length, and speed (median absolute errors ≤1%). Similar algorithm performances were obtained for all the other five cohorts in GE detection and propagation to the stride parameters, where an excellent absolute agreement with the pressure insoles was also found ( $ICC_{2,1}=0.817-\;0.999$ ). In conclusion, the proposed method accurately detects GE from marker data under different walking conditions and for a variety of gait impairments.

## Introduction

An individual’s ability to walk is usually quantified using spatio-temporal parameters (Perry and Davids, 2010; Preiningerova et al., 2015). Quantifying these parameters depends on the accurate identification of foot-to-ground events, namely, the initial (IC) and final (FC) contacts. Clinical gait analysis is traditionally performed during straight steady-state walking ( SW ), but it has been demonstrated that turning portions can also be informative for assessing gait impairments, especially in people with Parkinson’s disease (Crenna et al., 2007; El-Gohary et al., 2014; Curtze et al., 2016; Rehman et al., 2020; Shah et al., 2020) and in those at risk of falling (Bovonsunthonchai et al., 2015). Walking while turning is indeed included in different performance walk tests with either continuous walks over a fixed walking time (e.g., two–, six–, and twelve–minute walk tests (Butland et al., 1982)] or shorter walking tests with a fixed walking distance [i.e., Timed “Up and Go” (Nightingale et al., 2019) or its modified version, L-Test (Deathe and Miller, 2005)]. Similarly, gait parameters quantified during more complex gait-related activities, such as stair ascent, are sensitive in highlighting between-group differences not detected by clinical scales in various neurological diseases which cause mobility impairment (Carpinella et al., 2018). Therefore, quantifying walking ability while participants perform complex motor tasks might be preferred when aiming for a more discriminative assessment, particularly when evaluating patients in the early stages of their condition.

Foot-to-ground contacts can be accurately identified in laboratory settings using force platforms, which directly measure the exchanged forces (Bruening and Ridge, 2014; Lempereur et al., 2020), providing gold-standard temporal gait parameters. However, the number of consecutive gait events (GEs) is limited by the number of force platforms, their positioning, and by the correct foot positioning on them. This issue can be overcome when using foot switches or pressure insoles (PIs). When used as a standalone technology, none of the aforementioned tools, however, allow the direct quantification of spatial gait parameters, such as stride length or speed. Instrumented mats (e.g., GAITRite™, ProtoKinetics Zeno™, or Strideway™) can provide both spatial and temporal parameters (Van Uden and Besser, 2004), but only allow the analysis of straight walking and are not readily amenable to the use of walking aids. Moreover, the analysis is still restricted by their dimensions, and combining different mats can be very costly. Therefore, although still limited to a confined capture volume, the most suitable instruments for measurements of unconstrained gait spatio-temporal parameters during complex motor tasks in a laboratory setting are still marker-based stereophotogrammetric (SP) systems.

Optoelectronic stereophotogrammetry allows the tracking of the 3D position of retroreflective markers with high accuracy (<0.1 mm) and at a high sample rate (>100 Hz). GE identification from SP data can be obtained either manually or automatically. Previously proposed automatic GE detection algorithms, either based on peaks (Ghoussayni et al., 2004; Hsue et al., 2007; O’Connor et al., 2007; Zeni et al., 2008; Desailly et al., 2009), zero-crossing detection (Hreljac and Marshall, 2000), or machine learning (Filtjens et al., 2020; Lempereur et al., 2020) approaches, have been extensively tested on straight-line walking. Ulrich et al. (2019) recently tested some marker-based algorithms during turning, but only used a single force platform in different portions of a turn, which prevented the assessment of the complete turning maneuver and constrained turning location. To the authors’ knowledge, none of the marker-based methods have been tested across a variety of mobility tasks including potential confounding factors such as negotiating a step, turning, and sitting on a chair. Therefore, the aim of this study was to propose and validate a method for GE detection in rectilinear and curvilinear walking, and in step negotiation. The method’s performance was initially tested against existing methods using data from young healthy adults (YA). Its generalizability was then demonstrated using data from five cohorts, characterized by different gait patterns: healthy older adults (OA), patients with chronic obstructive pulmonary disease (COPD), multiple sclerosis (MS), Parkinson’s disease (PD), and proximal femur fracture (PFF). Finally, the propagation of temporal inaccuracies in GE detection on the quantification of spatio-temporal stride parameters was assessed.

## Materials and Methods

### Gait Event Detection Methods

**FIGURE 1:**
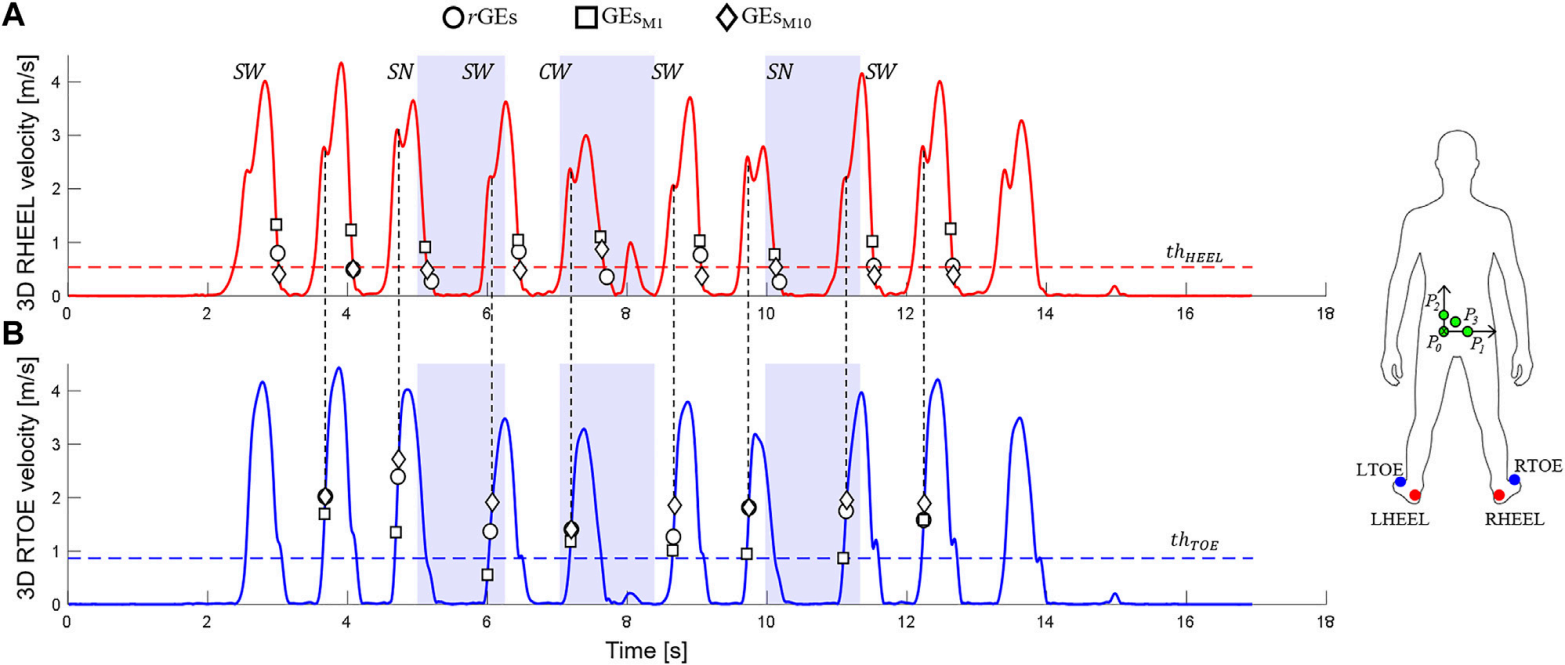
3D heel [in red, **(A)**] and toe [in blue, **(B)**] marker velocities (data from one participant performing a Hallway test, 2.3 section) are shown. Relevant ground-truth (circles, *r*GEs) and marker-based GEs (squares for the M1 method, GEs_M1_; diamonds for the M10 method, GEsM_10_) are indicated together with the adopted thresholds ( $th_{HEEL}$ and $th_{TOE}$ ). Straight-line walking ( SW ), curvilinear walking ( CW ), and step negotiation ( SN ) portions are highlighted with light blue rectangles. The figure also shows the adopted marker set (feet: LTOE, RTOE, LHEEL, and RHEEL; pelvic markers: P_0_, P_1_, P_2_, and P_3_) and pelvic axes [medio-lateral (ML) axis: unit vector going from P_0_ to P_1_, pointing to the right; anterior–posterior (AP) axis: unit vector orthogonal to the plane containing P_0_, P_1_, and P_3_ and pointing forward; vertical axis (V): unit vector orthogonal ML and AP and pointing cranially].

Ten methods for marker-based GE identification were evaluated in this study (Table 1). Among these, six methods, using either marker-trajectory positions (M1 and M2), velocities (M3 and M4), or accelerations (M8 and M9), were implemented as described in the literature (Hreljac and Marshall, 2000; Ghoussayni et al., 2004; Hsue et al., 2007; O’Connor et al., 2007; Zeni et al., 2008; Desailly et al., 2009). The main features of these methods, all using heel and toe markers (Figure 1), are summarized in Table 1. All methods except for M3 (O’Connor et al., 2007) used the anterior–posterior (AP) components of displacements, velocities, or accelerations. To this purpose, a reference system was identified in each frame (Cappozzo et al., 2005) using markers from a rigid cluster attached to the pelvis (Figure 1) and foot marker displacements, velocities, or accelerations were calculated along the three identified directions (anterior–posterior, AP; medio–lateral, ML; and vertical, V; Figure 1). Marker trajectories were filtered using a zero-lag fourth order Butterworth filter (cut-off frequency 7 Hz).

**TABLE 1:** Outline of the gait event identification methods adopted in this study.

Feature	Marker trajectory	References	Method	Component	Initial contact detection	Final contact detection
Position	*m*HEEL, *m*TOE, and *m*PELVIS	Zeni et al. (2008)	M1	AP	Local maxima of *m*HEEL_AP_ from *m*PELVIS	Local minima of *m*TOE_AP_ from *m*PELVIS
	*m*HEEL and *m*TOE	Desailly et al. (2009)	M2	AP	First maximum between high pass filtered *m*HEEL_AP_ and *m*TOE_AP_	Last minimum between high pass filtered *m*HEEL_AP_ and *m*TOE_AP_
Velocity	Mid-point between *m*HEEL and *m*TOE	O’Connor et al. (2007)	M3	V	Local minima	Local maxima
					*Additional constraints*: timing constraints, and threshold on vertical marker position	*Additional constraint*: timing constraints
	*m*HEEL and *m*TOE	Ghoussayni et al. (2004)	M4	AP and V	Sagittal *v*HEEL lower than 0.5 m/s^a^	Sagittal *v*TOE higher than 0.5 m/s^a^
		Enhanced M4	M5	3D	Rearfoot contacts: 3D *v*HEEL lower than 0.5 m/s^b^	3D *v*TOE higher than 1.0 m/s and then refined when the *v*HEEL decreases after its local peak^b^
					Forefoot contacts: 3D *v*TOE lower than 0.5 m/s	—
		M4 modified as suggested by Bruening and Ridge (2014)	M6	AP and V	Sagittal *v*HEEL lower than 0.78 * walking speed^c^	Sagittal vTOE higher than 0.66 * walking speed^c^
		Enhanced M6	M7	3D	Rearfoot contacts: 3D *v*HEEL lower than 0.5 * walking speed^b^	3D *v*TOE higher than 0.8 * walking speed^c^ and then refined when the *v*HEEL decreases after its local peak^b^
					Forefoot contacts: 3D *v*HEEL lower than 0.8 * walking speed^b^ ^,^ ^c^	—
Acceleration	*m*HEEL and *m*TOE	Hreljac and Marshall (2000)	M8	AP and V	Local maxima of *a*HEEL_V_	Local maxima of *a*TOE_AP_
					*Additional constraints*: relevant jerk is null	*Additional constraints*: relevant jerk is null
	*m*HEEL and *m*TOE	Hsue et al. (2007)	M9	AP and V	Local minima of *a*HEEL_AP_	Local maxima of *a*TOE_AP_
Position and velocity	*m*HEEL, *m*TOE, and *m*PELVIS	Combination of M1 and M7	M10	3D	Events initially detected using M1 and then refined according to the M7 conditions	Events initially detected using M1 and then refined according to the M7 conditions

a Velocity threshold increased from 0.1 m/s to 0.5 m/s as suggested in Bruening and Ridge ( 2014).

b Velocity thresholds adapted to the observed 3D velocities.

c Walking speed calculated for each test as the average stride speed; initial contacts detected with the method M5.

A modified version of M4, M6, was also implemented using an adaptive velocity threshold (Bruening and Ridge, 2014). The use of 3D rather than the marker velocity in the sagittal plane (AP-V plane) was also explored. In particular, candidate IC instances of time ( $t_{IC,H}$ ) were identified as those for which the magnitude of the 3D heel velocity vector was lower than 0.5 m/s (M5, fixed threshold) or 0.5*walking speed (M7, adaptive threshold). Since an IC might also occur with the forefoot (Bruening and Ridge, 2014), a further set of potential IC ( $t_{IC,T}$ ) was identified imposing a threshold on the 3D toe velocity vector magnitude [either fixed = 0.5 m/s (M5) or adaptive = 0.8*walking speed (M7)]. For each $t_{IC,H}$ , two checks were then performed:

1) If $t_{IC,H}$ ≤ $t_{IC,T}$ (rearfoot contact), then *mIC* = $t_{IC,H}$

2) If $t_{IC,H}$ > $t_{IC,T}$ , if the vertical position of the toe marker was lower than that of the heel at $t_{IC,T}$ (forefoot contact), then *mIC* = T ; else, (rearfoot contact) *mIC* = $t_{IC,H}$

For the FC detection (Figure 1), a threshold [fixed = 1.0 m/s (M5) or adaptive = 0.8*walking speed (M7)] was initially set on the 3D toe velocity. The instant ( $t_{FC,T}$ ) in which this threshold passed was used as the center of a 100 ms window within which a local peak of the magnitude of the 3D heel velocity vector was then sought. If this peak was found, indicating the initiation of the lifting of the foot (rotation around the ankle joint), then the *mFC* was set at the following instant (corresponding to a zero acceleration). If the peak was not found, then *mFC* = $t_{FC,T}$ (Figure 1).

A further new method, M10, was defined: estimations of both IC and FC were initially provided by M1, to reduce potential false positives exploiting the existence of markers on the pelvis, and then refined using the relevant events detected using M7. If the pelvic markers were occluded, the events were directly detected using M7.

Curvilinear walking ( CW ) was identified from the pelvis markers using simultaneous thresholds on the rotations around the V axis (≥45°) and the vertical angular velocity (peak ≥15°/s), with a constraint on duration ranging between 0.5 and 10 s (El-Gohary et al., 2014). Step negotiations ( SN ) were identified from the heel marker vertical displacement when the difference between consecutive ICs of the same foot was higher than 0.15 m. Each GE belonging to neither CW nor SN was labeled as included in straight-line walking ( SW ).

### Participants

A cohort of 20 YAs (Table 2) was recruited across two centers (University of Sheffield and University of Sassari) for the concurrent evaluation of the ten GE identification methods. All participants signed a consent form before taking part in the investigation (University of Sheffield Research Ethics Committee, Application number 029143).

**TABLE 2:** Demographic and clinical characteristics of the study participants.

Group	Subjects	Gender (% male)	Age [years]	BMI [kg/m^2^]	Clinical score	Walking pain	Walking aid users [n]
YA	20	55	29.6 (9.0)	23.2 (2.8)	N.A.	N.A.	0
OA	10	50	72.4 (6.8)	25.6 (4.2)	N.A.	1 (8)	0
						0–35	
COPD	10	50	72.1 (8.7)	25.5 (5.2)	CAT: 20 (15)	6 (14.5)	0
					Range: 6–31	0–43	
					FEV1/FVC%: 45% (20%)		
					Range: 21%–76%		
MS	10	60	53.1 (9.6)	31.9 (7.8)	EDSS: 4.5 (3.5)	3.5 (45)	3
					Range: 1.5–6.5	0–88	
PD	10	90	69.3 (6.0)	26.1 (4.3)	H&Y I: 2	6 (34)	0
					H&Y II: 6	0–45	
					H&Y III: 2		
PFF	10	70	82.9 (7.7)	24.3 (4.5)	SPPB: 8 (6)	4.5 (29.5)	1
					Range: 0–10	0–61	

Age [mean (standard deviation)], body mass index [BMI, mean (standard deviation)], clinical scores [median (interquartile range) and ranges or number of patients included in each category], and walking pain [median (interquartile range) and ranges] for the involved cohorts. CAT: chronic obstructive pulmonary disease (COPD) assessment test. EDSS, Expanded Disability Status Scale; H&Y, Hoehn and Yahr scale; YA, young healthy adults; OA, older adults; MS, multiple sclerosis; PD, Parkinson disease; PFF, proximal femur fracture; SPPB, short physical performance battery.

The generalizability of the results was then evaluated using a subset of the data from a multicentric study (Mobilise-D Technical Validation Study, Mazzà et al. [2021]), including 10 OAs, and 10 participants from the following four cohorts: COPD, MS, PD, and PFF. Participants (demographic and clinical characteristics shown in Table 2) were recruited from five centers and included in the study after providing written informed consent (Ethics approvals: Tel Aviv Sourasky Medical Center: the Helsinki Committee, 0551-19TLV; Robert Bosch Foundation for Medical Research: Medical Faculty of the University of Tübingen, 647/2019BO2; University of Kiel: Medical Faculty of Kiel University, D540/19; The Newcastle upon Tyne Hospitals NHS Foundation Trust and Sheffield Teaching Hospitals NHS Foundation Trust: London–Bloomsbury Research Ethics committee, 19/LO/1507). The adopted inclusion and exclusion criteria are detailed in Mazzà et al. (2021).

### Experimental Protocol

Reflective markers were attached bilaterally to participants’ shoes, in correspondence of the posterior side of calcaneus (HEEL) and of the second metatarsal head (TOE). Four markers were attached on the pelvis using a rigid cluster (Figure 1). The marker trajectories were acquired using different SP systems (8-camera Vicon T10, 10-camera Vicon T160, 12-camera Qualisys Miqus, 12-camera Vicon Vero, and 14-camera Vicon Bonita). Before data collection, a spot-check was performed to quantify the accuracy of the different SP systems, following the works of Scott et al. (2021). Pre-processing procedures were standardized with an ad-hoc pipeline, where foot trajectories were gap-filled only for gaps lower than 0.5 s (10.15131/shef.data.19115450). Participants were also equipped with a multi-sensing wearable system including two PIs, synchronized with the SP using a hardware-based solution (sampling frequency 100 Hz, Salis et al. [2021]).

Participants were asked to perform five structured mobility tasks (Mazzà et al., 2021):

• Straight-line walking: walk for 5 m at three self-selected speeds (slow, comfortable, and fast, twice each)
• Timed Up and Go: stand-up from a chair, walk for 3 m, turn around (U-turn, ∼ 180°), walk back to the chair, and sit down
• L-Test: stand-up from a chair, walk for 4 m, turn 90° to the left, walk for 2 m, U-turn to the left (∼180°), walk back, turn 90° to the right, walk 2 m back to the chair, and sit down
• Surface test: walk twice in a loop (∼4 m rectilinear, and four ∼180° U-turns), with different surfaces along the path
• Hallway test: walk 6 m, stepping up and down a step, turn 180° turn, and walk back (again negotiating the step)

### Data Processing and Statistical Analysis

The PI signals were used to isolate the different walking bouts [defined as comprising of at least two right and two left strides (Kluge et al., 2021)] and all reference GEs (*r*GEs) were identified according to the methodology proposed and validated by Salis et al. (2021).

The ten GE methods were compared using the YA data and the following performance criteria:

• Sensitivity (S), positive predictive values (PPV), and F1 values: for each *r*GE, a marker-based GE was classified as a true positive (TP), false negative (FN), or false positive (FP) using a tolerance window (TW) of 0.5 s centered on rGE.

$$S_{M,W}=\frac{TP_{M,W}}{TP_{M,W}+FN_{M,W}},$$ (1)

$$PPV_{M,W}=\frac{TP_{M,W}}{TP_{M,W}+FP_{M,W}},$$ (2)

$$F_{1\;M,W}=2*\frac{PPV_{M,W}*S_{M,W}}{PPV_{M,W}+S_{M,W}},$$ (3)

where M = M1,…, M10 are the different methods, and W = SW, CW, SN are the walking conditions. When foot marker occlusions prevented the identification of a GE, the corresponding *r*GEs were excluded from the analysis.

• Accuracy, bias, and precision: for each identified TP, the relevant time error of a method M in condition W ( $\Delta t_{M,W}$ ) was characterized using median absolute errors ( $MAE_{M,W}$ ), median errors ( $ME_{M,W}$ ), and inter-quartile range errors ( $IQRE_{M,W}$ ) which are used to establish the relevant accuracy, bias, and precision, respectively, as suggested in the work of Walther and Moore (2005).

Reference stride, stance, and swing phase durations were quantified using the *r*GEs, and foot marker trajectories were used to calculate the reference length and speed during these time intervals. The impact of the GE detection inaccuracies on all other parameters was then assessed for each method and condition. The errors were computed only for the strides identified by TP ICs, with the remaining strides counted as missing. TP strides were further labeled as curvilinear or step negotiation strides if they had at least one IC belonging to either CW or SN , respectively. Accuracy, bias, and precision, both absolute and relative, were calculated for all parameters as previously described for the GEs.

The aforementioned analyses allowed the method that best satisfied the GE performance criteria to be chosen. Its generalizability was then established by applying it to the data from the five different cohorts and repeating all the aforementioned analyses, both at the event level and stride level.

Kolmogorov–Smirnov tests were used to assess the normality of the error distributions for the $\Delta t_{M,W}$ in the YA group. A Friedman test assessed differences in performance among the methods for identification of both ICs and FCs under all walking conditions and a Wilcoxon signed–rank post hoc test evaluated pairs of methods, using a Bonferroni Holm’s correction for multiple comparisons. For the statistical analysis, FN events were assigned the highest error observed for each adopted method when more than 5% of the expected errors were missing or the mean error values otherwise (Scheffer, 2002). FP events were not included in the analysis.

For all cohorts, Bland–Altman (BA) plots (Martin Bland and Altman, 1986) were used to visually compare the marker-based parameters and check for nonlinear or heteroscedastic distributions of the differences between them. Absolute agreements were tested using intraclass correlation coefficients ( $ICC_{2,1}$ ) and their 95% confidence intervals [absolute-agreement, two-way mixed-effects model, Koo and Li (2016)] and relative agreement using Spearman correlation coefficients. Limits of agreement (LoA) and root mean square errors (RMSE) were calculated for the TP strides parameters of each cohort. All analyses were conducted using SPSS (version 26-IBM SPSS Inc., Chicago, United States ) and statistical significance for all tests was set to *p* < 0.05.

## Results

### Selection of the Best Gait Event Detection Method

**FIGURE 2:**
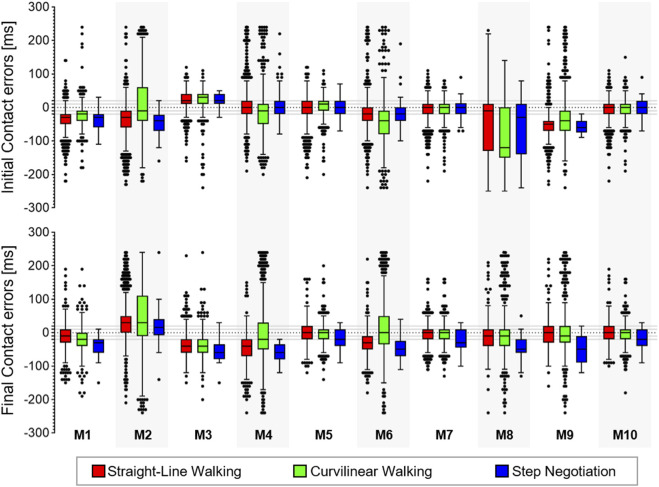
Box-plots (minimum, lower quartile, median, upper quartile, and maximum) of the error (ms) for the true positive (TP) initial and final contacts from the 10 methods (M1, … , M10) in the young healthy adult cohort in the three walking conditions. Outliers are also shown.

Overall, 4,476 GEs (2,427 ICs) were detected with the PIs for the YA cohort, of which 2,876 were in SW , 1,468 in CW, and 132 in SN . For each method M and walking condition W , Table 3 shows sensitivity, PPV, F1 scores, and performance metrics for the different methods for both GEs, while Figure 2 shows the distribution of the temporal inaccuracies ( $\Delta t_{M,W}$ ).

**TABLE 3:** Performances of the 10 methods (M1 … M10) in detecting both initial and final contacts in the young healthy adult cohort in straight-line walking ( SW ), curvilinear walking ( CW ), and step negotiation ( SN ), reported in terms of sensitivity ( S ), positive predictive values (PPV), F1 scores, median error (ME, i.e., bias), inter-quartile range error (IQRE, i.e., precision), and median absolute error (MAE, i.e., accuracy). S , PPV, and F1 values lower than 85% have been highlighted in italic.

		Straight-line walking						Curvilinear walking						Step negotiation					
		S (%)	PPV (%)	F1 (%)	ME (ms)	IQRE (ms)	MEA (ms)	S (%)	PPV (%)	F1 (%)	ME (ms)	IQRE (ms)	MAE (ms)	S (%)	PPV (%)	F1 (%)	ME (ms)	IQRE (ms)	MAE (ms)
**Initial contacts**	M1	99.1	99.8	99.4	−30	30	30	98.8	97.7	98.3	−20	30	30	100.0	100.0	100.0	−30	40	30
	M2	96.3	*72.9*	*83.0*	−30	50	30	93.9	*51.9*	*66.8*	−10	100	50	97.0	98.5	97.7	−40	45	40
	M3	98.0	96.9	97.4	20	30	30	94.7	97.2	96.0	30	30	30	*54.5*	97.3	*69.9*	20	30	25
	M4	99.3	*71.6*	*83.2*	0	40	20	*79.5*	*52.3*	*63.1*	−10	60	30	100.0	94.4	97.1	0	40	20
	M5	99.3	99.9	99.6	0	40	20	99.5	99.2	99.3	10	30	20	100.0	100.0	100.0	0	40	20
	M6	99.4	*79.6*	88.4	−20	40	20	*84.6*	*47.4*	*60.8*	−40	70	40	100.0	93.1	96.4	−20	40	20
	M7	99.3	99.9	99.6	0	30	10	99.5	99.3	99.4	0	30	20	100.0	100.0	100.0	0	30	20
	M8	97.3	*39.2*	*55.9*	−10	140	20	98.9	*41.8*	*58.8*	−120	150	120	98.5	*65.7*	*78.8*	−30	150	40
	M9	*78.2*	*79.3*	*78.7*	−50	30	60	*70.9*	*43.9*	*54.2*	−40	57.5	50	*43.1*	96.2	*59.5*	−60	33	60
	M10	99.3	100.0	99.7	0	30	10	99.3	99.9	99.6	0	30	20	100.0	100.0	100.0	0	30	20
**Final contacts**	M1	99.8	99.8	99.8	−10	40	20	97.5	96.2	96.8	−20	40	30	100.0	100.0	100.0	−30	40	30
	M2	97.8	*36.7*	*53.4*	30	50	40	96.2	*31.3*	*47.3*	30	120	60	97.0	*64.0*	*77.1*	15	45	30
	M3	98.8	99.4	99.1	−40	40	40	97.8	99.0	98.4	−40	40	40	100.0	100.0	100.0	−60	43	60
	M4	99.5	*64.4*	*78.2*	−40	50	40	*81.4*	*42.6*	*55.9*	−20	80	50	100.0	89.2	94.3	−60	43	60
	M5	98.9	100.0	99.5	0	40	20	97.8	99.9	98.8	0	30	20	100.0	100.0	100.0	−20	50	30
	M6	99.8	*69.6*	*82.0*	−30	40	30	*81.5*	*40.1*	*53.7*	0	83	40	100.0	91.7	95.7	−50	43	50
	M7	98.9	100.0	99.5	0	30	20	98.1	99.9	99.0	0	30	20	100.0	100.0	100.0	−30	53	30
	M8	*82.8*	*75.1*	*78.8*	−10	50	20	*76.8*	*37.5*	*50.4*	−10	50	30	*60.0*	94.7	*73.5*	−50	40	50
	M9	*76.5*	85.1	*80.6*	0	50	20	*66.1*	*42.6*	*51.8*	−10	50	30	*20.7*	100.0	*34.3*	−50	60	50
	M10	100.0	99.9	100.0	0	40	20	99.0	99.6	99.3	0	30	20	100.0	100.0	100.0	−20	50	30

For each walking condition W , the Friedman tests highlighted significant differences in $\Delta t_{M,W}$ values for both GEs (*p* < 0.001) and pairwise comparisons showed that the newly proposed methods M5, M7, and M10 outperformed most of the others (Supplementary Table S1 and Supplementary Table S2). Since M10 also had the highest F1 scores under all walking conditions for both GEs, it was selected as the best performing method. Using M10, the gait events were identified with a 40 ms (4 frames) accuracy ranged from 89% ( SW ) to 91% ( CW ) for ICs and from 86% ( SW ) to 89% ( SW ) for FCs.

### Propagation of Gait Event Inaccuracies on the Estimate of the Stride Level Parameters

Overall, 1,000, 981, and 89 strides were detected for the YA cohort in SW , CW, and SN , respectively. Bias, precision, and accuracy for the stride parameter errors for the ten methods are reported in Supplementary Table S3. For the best performing method (M10), the median absolute percentage error (i.e., accuracy) was ≤1% for the stride duration, length, and speed under all walking conditions, except for the SN stride duration (1.9%). For stance and swing duration, absolute accuracy errors were similar to those of the stride duration, but caused larger relative accuracy errors (between 2.4% for stance duration in both SW and CW , and 7.1% in SN ). Around 20% of the TP strides had errors in length lower or equal to the accuracy of the two SP systems (linear RMSE = 1.2 mm). Overall, stride length errors had virtually no bias and an MAE ≤ 0.5% for both SW and CW ; a MAE≤ 1% was observed in SN , resulting from the observed temporal inaccuracies. About a quarter of the detected strides had duration errors equal to or lower than the system temporal resolution ( $\text{Δ}t_{min}=$ 10 ms for a sampling frequency of 100 Hz). The same applied to 15.2% of the stance and 20.9% of the swing phase errors. Almost 70% (69.2%) of TP strides had speed errors equal to or lower than 0.01 m/s.

### Accuracy of M10 in Pathological Gait

**FIGURE 3:**
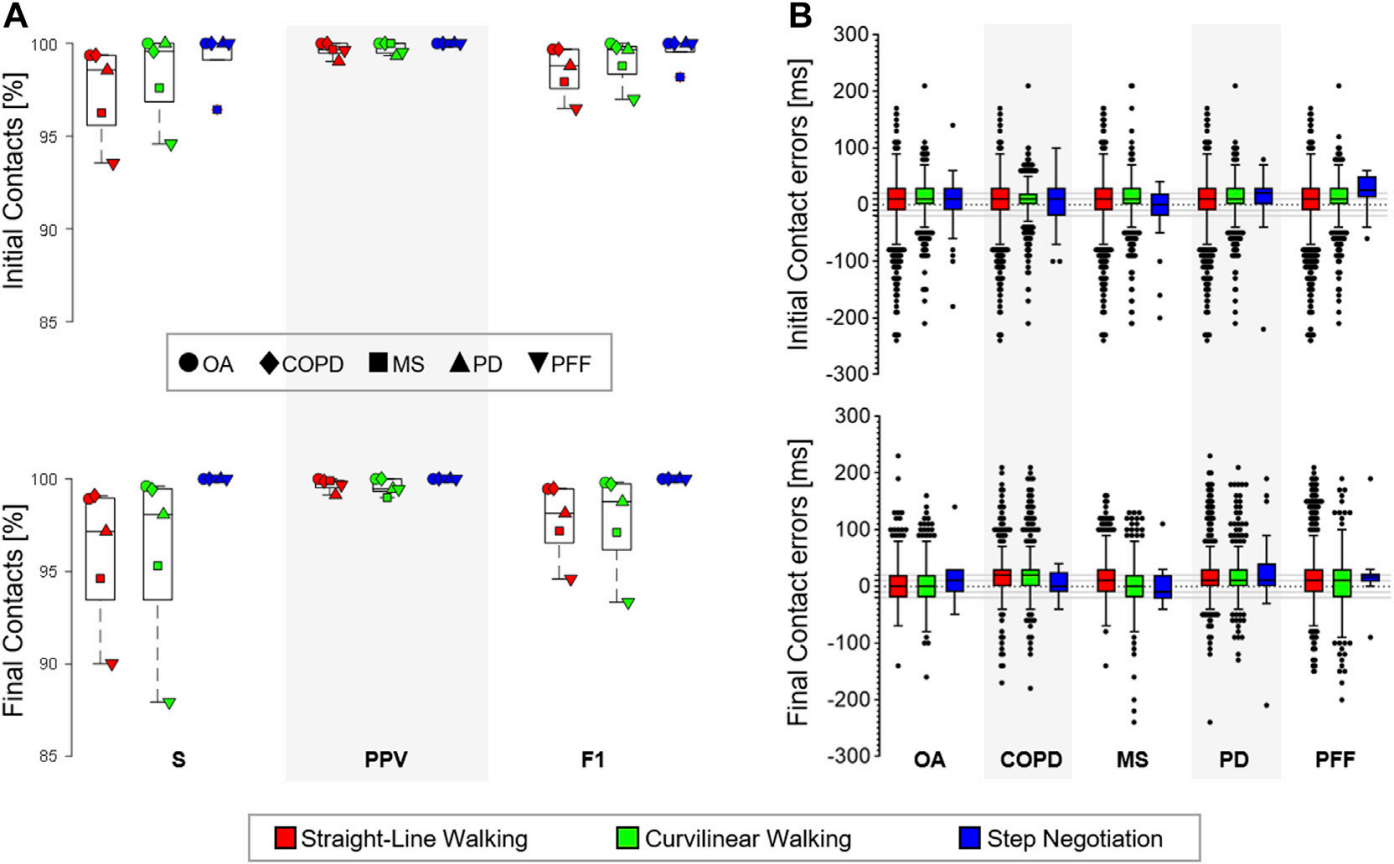
**(A)** Sensitivity ( S ), positive predictive values (PPV), and F1 scores observed using the selected method in the older adults (OA), chronic obstructive pulmonary disease (COPD), multiple sclerosis (MS), Parkinson disease (PD), and proximal femur fracture (PFF) cohorts in the three walking conditions: straight-line walking, curvilinear walking, and step negotiation. **(B)** Box-plots (minimum, lower quartile, median, upper quartile, and maximum) of the error for the TP initial and final contacts (ms) observed using the selected method in the five (OA, COPD, MS, PD, and PFF) cohorts during the three walking conditions. Outliers are also shown.

Overall, 2,514 (1,337 ICs) gait events were detected for the OA cohort, 3,172 (1,681 ICs) for the COPD, 3,548 (1,879 ICs) for the MS, 2,766 (1,468 ICs) for the PD, and 3,042 (1,609 ICs) for the PFF cohorts. Figure 3 shows the sensitivity, PPV, F1 scores, and performance metrics for M10 in the three walking conditions. The IC events identified within four frames (40 ms) ranged between 65% ( SN , PFF) and 93% ( SW , PD) and the FCs between 75% ( CW , PFF) and 100% ( SN , COPD).

The overall detected strides were 1,174 in the OA (39% in SW , 55% in CW, and 6% in SN ), 1,483 in the COPD (36% in SW , 59% in CW, and 5% in SN ), 1,582 in the MS (49% in SW , 49% in CW, and 2% in SN ), 1,294 in the PD (37% in SW , 58% in CW, and 5% in SN ), and 1,315 in the PFF cohorts (59% in SW , 39% in CW, and 2% in SN ). Bias, precision, and accuracy of the errors for the stride-level parameters are reported in Table 4. A 10 ms bias (1 frame delay) was observed in the IC identification for most cohorts and walking conditions (Figure 3). In most cases, this error propagated with a virtually null bias in the stride duration (Table 4).

**TABLE 4:** Correctly detected strides (%) for each cohort (young adults (YA), older adults (OA), multiple sclerosis (MS), Parkinson disease (PD), chronic obstructive pulmonary disease (COPD); and proximal femur fracture (PFF) and walking conditions (straight-line walking, SW ; curvilinear walking, CW ; and step negotiation, SN ) are reported. The errors in the relevant stride duration (ms), length (mm), speed (mm/s), and stance/swing durations (ms) are described in terms of median (ME, *i.e.,* bias), inter-quartile range (IQRE, *i.e.,* precision), and median absolute errors (MAE, *i.e.,* accuracy); relative errors (%) are also shown.

			Stride duration error						Stride length error						Stride speed error						Stance duration error						Swing duration error					
			ME		IQRE		MAE		ME		IQRE		MAE		ME		IQRE		MAE		ME		IQRE		MAE		ME		IQRE		MAE	
Strides (%)			( ms )	(%)	( ms )	(%)	( ms )	(%)	( mm )	(%)	( mm )	(%)	( mm )	(%)	( $\frac{mm}{s}$ )	(%)	( $\frac{mm}{s}$ )	(%)	( $\frac{mm}{s}$ )	(%)	( ms )	(%)	( ms )	(%)	( ms )	(%)	( ms )	(%)	( ms )	(%)	( ms )	( ms )
*SW*	YA	98.5	0	0.0	30	2.0	10	0.9	−0.8	−0.1	10.3	0.8	5.8	0.5	0.6	0.1	9.6	0.9	4.5	0.4	−10	−1.1	40	4.3	20	2.4	0	0	30	7.4	20	4.3
	OA	100.0	0	0.0	30	2.3	10	1.0	−0.5	0.0	12.3	1.1	6.2	0.6	0.2	0.0	10.6	1.1	4.9	0.5	0	0	30	4.4	20	2.5	−10	−2.4	40	9.8	20	5.1
	COPD	100.0	0	0.0	20	1.8	10	0.9	0.1	0.0	12.0	1.0	5.9	0.5	0.0	0.0	9.6	1.0	4.9	0.5	0	0	40	4.9	20	2.5	0	0	32.5	8.5	20	5
	MS	95.7	0	0.0	30	2.0	10	0.9	−0.1	0.0	9.9	1.0	5.0	0.5	0.0	0.0	7.8	1.0	3.9	0.5	0	0	50	5.1	20	2.5	0	0	40	10.3	20	5.1
	PD	99.2	0	0.0	30	2.1	10	0.9	−0.2	0.0	10.2	1.0	5.1	0.5	0.3	0.0	7.8	0.9	4.0	0.5	−10	−1	60	6.1	30	3.2	0	0	50	11.5	30	5.8
	PFF	94.3	0	0.0	40	2.4	20	1.2	−0.4	0.0	9.5	1.3	4.9	0.6	−0.1	0.0	6.6	1.2	3.4	0.6	0	0	60	7	30	3.4	0	0	60	16	30	8
*CW*	YA	98.4	0	0.0	20	1.8	10	0.9	0.2	0.0	12.6	1.0	6.3	0.5	0.0	0.0	12.3	1.2	6.1	0.6	0	0	40	5	20	2.5	0	0	40	9.8	20	4.8
	OA	99.8	0	0.0	20	2.0	10	1.0	1.0	0.1	13.9	1.4	6.8	0.7	0.0	0.0	10.6	1.1	5.4	0.6	10	1.3	40	5.3	20	2.9	−10	−2.6	40	10.9	20	5.3
	COPD	99.3	0	0.0	20	1.7	10	0.9	0.0	0.0	12.0	1.1	5.9	0.6	−0.2	0.0	8.7	1.0	4.5	0.5	0	0	40	4.9	20	2.4	0	0	40	10	20	4.9
	MS	95.7	0	0.0	20	1.8	10	0.9	0.0	0.0	10.7	1.1	5.4	0.6	0.0	0.0	9.5	1.3	4.7	0.6	10	1.3	50	5.2	30	2.8	−10	−2.6	50	13.4	30	7
	PD	100.0	0	0.0	20	1.7	10	0.9	−0.3	0.0	12.3	1.3	6.0	0.6	−0.3	0.0	8.0	1.0	4.0	0.5	0	0	40	5	20	2.5	0	0	40	9.3	20	4.7
	PFF	91.4	0	0.0	40	2.9	20	1.5	−0.1	0.0	17.0	2.3	8.1	1.1	−0.2	−0.1	10.0	1.6	4.9	0.8	20	2.2	70	7.4	40	4.3	−20	−5.1	60	16	30	9.3
*SN*	YA	100.0	−10	−0.7	40	3.7	20	1.9	6.6	0.5	23.5	1.9	13.0	1.0	7.8	0.0	28.3	1.2	16.4	0.6	0	0	40	4.9	20	2.4	−10	−2.4	50	13	30	7.1
	OA	100.0	0	0.0	58	4.1	30	1.9	−0.5	0.0	22.6	1.8	9.9	0.8	−3.6	−0.4	28.2	3.4	10.3	1.2	−10	−0.9	50	7	30	3.2	0	0	50	12	30	6.5
	COPD	100.0	0	0.0	55	4.3	30	2.3	2.3	0.2	29.7	2.6	14.3	1.3	−0.9	−0.1	22.7	2.6	10.1	1.3	−10	−1	50	6	25	3.1	10	2.6	60	12.2	30	6.5
	MS	97.3	10	0.7	50	2.7	20	1.6	−0.4	−0.1	11.3	1.3	5.7	0.6	−2.8	−0.4	9.3	1.9	5.4	0.8	10	0.7	57.5	5.6	30	3	−10	−1.6	40	9.9	20	5.1
	PD	100.0	0	0.0	30	2.6	20	1.5	−0.2	0.0	26.8	2.5	14.0	1.3	−0.4	0.0	13.4	1.6	6.5	0.8	0	0	55	6.6	20	2.1	15	3	75	15.1	30	7.3
	PFF	100.0	−10	−0.7	27	1.7	20	1.2	−5.5	−0.6	26.4	4.0	10.7	1.3	−2.8	−0.4	13.4	2.8	7.9	1.4	10	1.4	55	4.4	20	2.1	−20	−4.8	50	15.5	30	6.8

In light of the spatial resolutions assessed for each cohort, virtually null errors in the stride length were observed in 14.7%, 10.5%, 25.4%, 23.5%, and 12.8% of the cases for the OA (<1.5 mm), COPD (<0.6 mm), MS (<2.0 mm), PD (<1.3 mm), and PFF (<0.8 mm) cohorts, respectively. Similarly, the percentage of strides in which the errors in the stride duration were equal to or lower than the temporal resolution ( $\text{Δ}t_{min}$ ) ranged between 18.5% (PFF) and 24.1% (MS), those for the stance duration between 8.3% (PFF) and 16.4% (OA), and those for the swing duration between 9.5% (PFF) and 17.8% (PD). Finally, more than 70% of the errors in the stride speed were equal to or lower than 0.01 m/s in all cohorts (OA: 71.3%, COPD: 76.6%, MS: 77.3%, PD: 77.9%, and PFF:71.3%).

**FIGURE 4:**
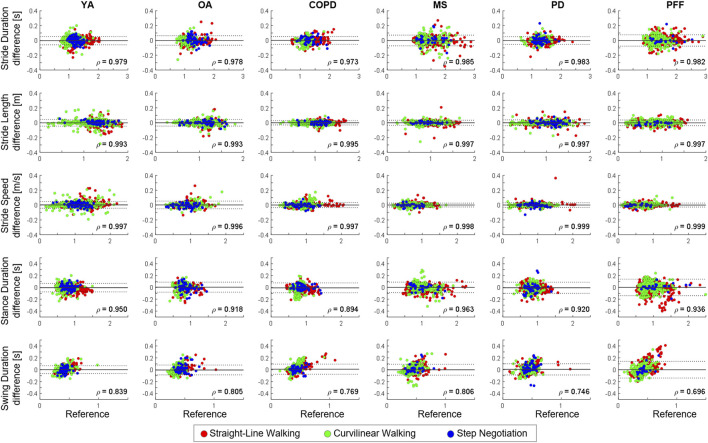
Bland–Altman (BA) plots of the different stride-level parameters [stride length (m), stride duration (s), stride speed (m/s), and stance/swing duration (s)] in the young healthy adults (YA), older adults (OA), chronic obstructive pulmonary disease (COPD), multiple sclerosis (MS), Parkinson disease (PD), and proximal femur fracture (PFF) cohorts. Strides detected during straight-line walking, curvilinear walking, and step negotiation are reported in green, red, and blue, respectively. In each BA plot, bias (mean value, gray line) and limits of agreements (bias ±1.96 standard deviations; black with dotted lines) are represented. Spearman correlation coefficients ( ρ ) are also shown, all ρ values were statistically significant (*p* < 0.001).

For the TP strides, the relevant errors for each cohort are shown using Bland–Altman plots; errors for the YA group are also provided in (Figure 4). Excellent absolute ( $ICC_{2,1}$ >0.9) and relative agreement ( ρ>0.9 ) were observed in all cohorts and for most of the stride-related parameters, except for swing duration (Table 5; Figure 4). For the latter parameter, a heteroscedastic distribution of the error can be observed, especially in the PFF cohort (Figure 4).

**TABLE 5:** For each stride parameter (stride duration, length, speed, and stance/swing duration) and cohort (young healthy adults (YA), older adults (OA), chronic obstructive pulmonary disease (COPD), multiple sclerosis (MS), Parkinson disease (PD), and proximal femur fracture (PFF), root mean square error (RMSE) values, limits of agreement (LOA), ICC_2,1_ with its 95% confidence interval (CI) are shown.

		RMSE	LOA	ICC_2,1_	95% ICC_2,1_ CI
Stride duration (ms)	YA	29	(−59 – 54)	0.994*	(0.993–0.994)
	OA	33	(−65 – 63)	0.992*	(0.991–0.993)
	COPD	27	(−53 – 53)	0.993*	(0.992–0.994)
	MS	37	(−74 – 71)	0.995*	(0.995–0.996)
	PD	26	(−53 – 51)	0.995*	(0.994–0.995)
	PFF	39	(−78 – 76)	0.995*	(0.994–0.996)
Stride length (mm)	YA	23	(46 – 44)	0.997*	(0.997–0.997)
	OA	23	(−45 – 44)	0.997*	(0.997–0.998)
	COPD	15	(−30 – 28)	0.999*	(0.998–0.999)
	MS	17	(−35 – 33)	0.998*	(0.998–0.999)
	PD	20	(−40 – 38)	0.999*	(0.998–0.999)
	PFF	20	(−40 – 37)	0.999*	(0.999–0.999)
Stride speed (mm/s)	YA	23	(−43 – 48)	0.998*	(0.998–0.998)
	OA	27	(−51 – 53)	0.997*	(0.996–0.997)
	COPD	18	(−34 – 34)	0.999*	(0.998–0.999)
	MS	13	(−26 – 26)	0.999*	(0.999–0.999)
	PD	16	(−31 – 32)	0.999*	(0.999–0.999)
	PFF	14	(−27 – 27)	0.999*	(0.999–0.999)
Stance duration (ms)	YA	37	(−76 – 67)	0.984*	(0.982–0.985)
	OA	43	(−81 – 88)	0.974*	(0.971–0.977)
	COPD	43	(−92 – 75)	0.967*	(0.962–0.972)
	MS	45	(−89 – 88)	0.999*	(0.999–0.999)
	PD	48	(−101 – 88)	0.970*	(0.966–0.973)
	PFF	71	(−140 – 139)	0.978*	(0.975–0.980)
Swing duration (ms)	YA	34	(−65 – 68)	0.922*	(0.914–0.928)
	OA	43	(−88 – 79)	0.900*	(0.888–0.911)
	COPD	45	(−77 – 94)	0.883*	(0.867–0.898)
	MS	47	(−92 – 91)	0.990*	(0.989–0.991)
	PD	49	(−89 – 100)	0.876*	(0.861–0.889)
	PFF	72	(−140 – 140)	0.817*	(0.795–0.836)

*p < 0.0001.

## Discussion

This study aimed to propose a method for marker-based gait event detections from motion capture data in complex motor tasks and demonstrate its applicability to gait assessment in different conditions. Using reference gait events detected with pressure insoles, several methods were initially compared with data collected from young healthy adults and the best results were achieved by combining a method based on the AP trajectories (Zeni et al., 2008), largely used in the literature and already tested on different populations (Zeni et al., 2008; Bruening and Ridge, 2014; Hendershot et al., 2016; Filtjens et al., 2020; Gonçalves et al., 2020; Lempereur et al., 2020; Visscher et al., 2021), with an innovative solution exploiting 3D foot velocities, which overcame previously reported issues associated with gait event anticipation. This method (M10) provided estimations with a virtually null bias for both initial and final contacts for all investigated variables, except for a 20 ms bias (2 frame anticipation) for the final contact detection during step negotiation. Very few GEs were missed and extra events were introduced, as shown by the very high values of both sensitivity and PPV (>99% overall). Additionally, F1 scores higher than 99% were recorded in all the three walking conditions, confirming the method is able to correctly identify GEs.

From a methodological perspective, the fact that M10 was the best method is supported by the previous literature using feet marker velocity features (Bruening and Ridge, 2014; Gonçalves et al., 2020; Visscher et al., 2021). When using only the sagittal velocity as per previous literature (M4/M6), a very high sensitivity was observed in the absence of changes of direction (straight-line walking or step negotiation). However, the performance of M4/M6 clearly deteriorated when investigating turning, as previously reported in both young and older participants (Ulrich et al., 2019). This was true also when accounting for changes of direction using the pelvis reference system, likely due to the turn initiation of the foot being delayed with respect to that of the pelvis (Akram et al., 2010). Using 3D velocity overcame this issue, justifying the better results obtained for both M7 and M10.

Having demonstrated superior performance in terms of higher sensitivity and positive predicted values, the generalizability of M10 was then tested on data from five other cohorts, including older adults and patients suffering from conditions regularly associated with distinct gait impairment. High F1 scores (>95%) were still observed for all walking conditions and cohorts, with the only exception of the GEs in the PFF cohort, where for patients with the highest disability (SPPB score ≤4) some GEs were missed in both straight-line and curvilinear walking. Generally, extra and missing GEs were observed in patients using walking aids, reporting severe walking pain, or having the highest disability scores, suggesting that a visual check of the data should always be performed in patients with severely affected gait for data veracity. A null bias was observed in 20% of the observed cases (cohorts and walking conditions) for IC and FC and a residual bias ≤20 ms in all others. Considering previous literature indicates an accuracy of 21 ms for the pressure insoles (Salis et al., 2021), these residual biases can be considered entirely negligible for the ICs. However, they might still need to be accounted for when investigating FCs, where the insoles have an average error of 3 ms (Salis et al., 2021); it is unlikely that such a small difference has a practical relevance. Overall, reported results clearly show that the newly proposed M10 method can be used to accurately extract GEs under different walking conditions and in the presence of a variety of gait impairments.

Visscher et al. (2021) recently quantified how the temporal inaccuracies associated with the detection of gait events propagate to other spatio-temporal parameters, reporting relevant effects only on step width and single limb support. These results were confirmed here in SW for all cohorts investigated. In this study, virtually zero bias and very satisfactory precision values were observed (IQR error values ranging between 0.8% for stride length in YA and 2.4% for the stride duration in PFF). The latter finding is comparable to the maximal limits of agreement (−3% to 4%) reported by Visscher et al. (2021) for the stride length error in children with cerebral palsy. The same was true for the swing phase: precision error from 7.4% (YA) to 16.0% (PFF), which was again similar to the single limb support limits of agreement (−12% to 16%) observed in Visscher et al. (2021). Slightly bigger effects in terms of error propagation were observed in SN and CW , where the bias remained virtually zero for all cohorts, but the IQR error reached 4.3% for step negotiation duration in OA and COPD. For swing duration, the error had a heteroscedastic behavior (Figure 4), especially in PFF. This was likely due to errors in final contact identifications. Further studies are needed to establish whether these propagated inaccuracies in SN duration and swing duration lead to clinically meaningful differences when investigating complex tasks in PFF. Overall, the excellent absolute ( $ICC_{2,1}$ >0.9, $ICC_{2,1}$ range <0.001 in all cases, except swing duration in PD and PFF, Table 5) and relative (ρ > 0.9, in all the cases except swing duration, Figure 4) agreement have been observed in the explored stride parameters confirming the suitability of the method for the investigated cohorts.

This study has some limitations. First, the cohorts were too small to include approaches based on machine learning [e.g., Filtjens et al. (2020) and Lempereur et al. (2020)] in the comparison. Nonetheless, the very satisfactory results obtained with M10 seem to leave little room for improvement. Second, the number of events and strides investigated in SN and CW were much lower than those in SW . Although they were sufficient for the analysis reported here, an even stronger validation might focus on assessing the accuracy of the methods in different types of turnings (e.g., sharp versus larger turns) or step ascending/descending tasks (e.g., multiple steps). Finally, one constraint on a wide adoption of the proposed method is the use of a cluster of markers at the pelvis which are not part of standard gait analysis protocols, unlike the foot markers. Although the method can be easily implemented using a reference system built from skin markers on the pelvis, the potential differences associated with pelvic soft tissue artifacts (Camomilla et al., 2017) that might affect the initial gait event estimations (M1) should be mitigated when those events are refined exploiting the foot velocity information. Nevertheless, future studies including participants with high BMIs, both pelvic marker sets and reference gait events are needed to confirm this assumption.

In conclusion, the proposed strategy can be combined with motion capture data to automatically extract accurate gait events during complex motor tasks in both young and older healthy individuals and in patients with PD, MS, COPD, and PFF. As an example of a possible application, the method is currently being used as part of a multi-centric study where different stereophotogrammetric systems are used as the gold standard for the validation of digital mobility outcomes obtained from a single inertial sensor device attached to the pelvis (Mazzà et al., 2021). To foster its adoption, the methodology implemented in the present study has been made available *via* Figshare (https://doi.org/10.15131/shef.data.19102619.v1).

## Data Availability

The datasets presented in this study can be found in online repositories. The names of the repository/repositories and accession number(s) can be found at: https://doi.org/10.15131/shef.data.19102619.v1.
