# Supplementary material for: An Algorithm for Accurate Marker-Based Gait Event Detection in Healthy and Pathological Populations During Complex Motor Tasks
Source: Front Bioeng Biotechnol. 2022 Jun 2;10:868928. doi: 10.3389/fbioe.2022.868928 (PMC9201978; doi:10.3389/fbioe.2022.868928)
Supplement: Supplementary file 1 [file Table1.DOCX]

Supplementary Material

# Supplementary results

## Selection of the best gait event detection method

**Supplementary Table 1.** Post hoc analysis of all the pairwise comparisons of the error distribution ($\Delta t$) observed for the different initial contact detection methods (M1… M10) in the three walking conditions: straight-line walking ($SW$), curvilinear walking ($CW$) or a step negotiation ($SN$). All the p values have been reported in the table; when a statistical significant difference in the relevant $\Delta t$pairwise comparison was observed (p < 0.05), the best performing method has been indicated.


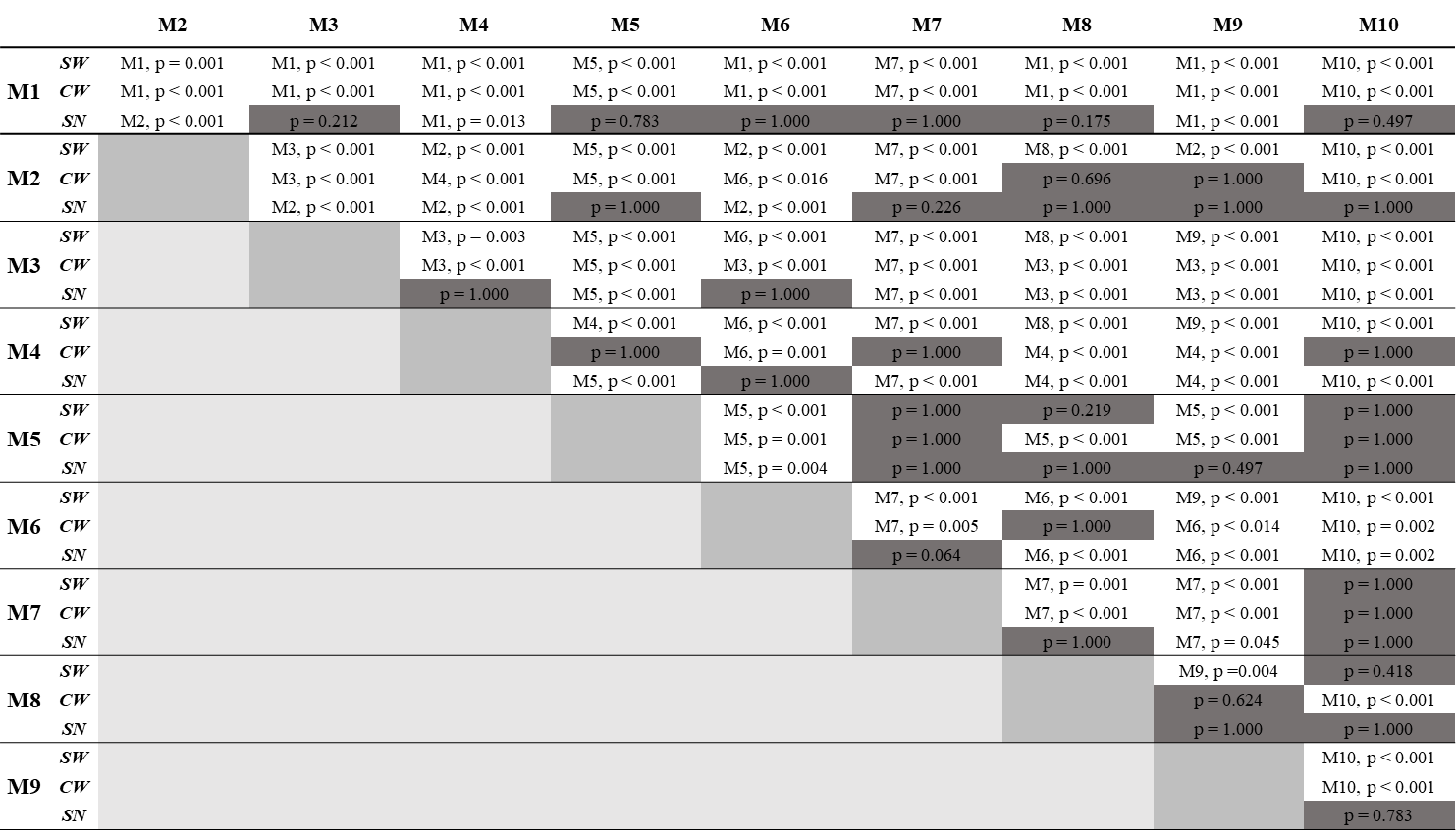


**Supplementary Table 2.** Post hoc analysis of all the pairwise comparisons of the error distribution ($\Delta t$) observed for the different final contact detection methods (M1… M10) in the three walking conditions: straight-line walking ($SW$), curvilinear walking ($CW$) or a step negotiation ($SN$). All the p values have been reported in the table; when a statistical significant difference in the relevant $\Delta t$pairwise comparison was observed (p < 0.05), the best performing method has been indicated.


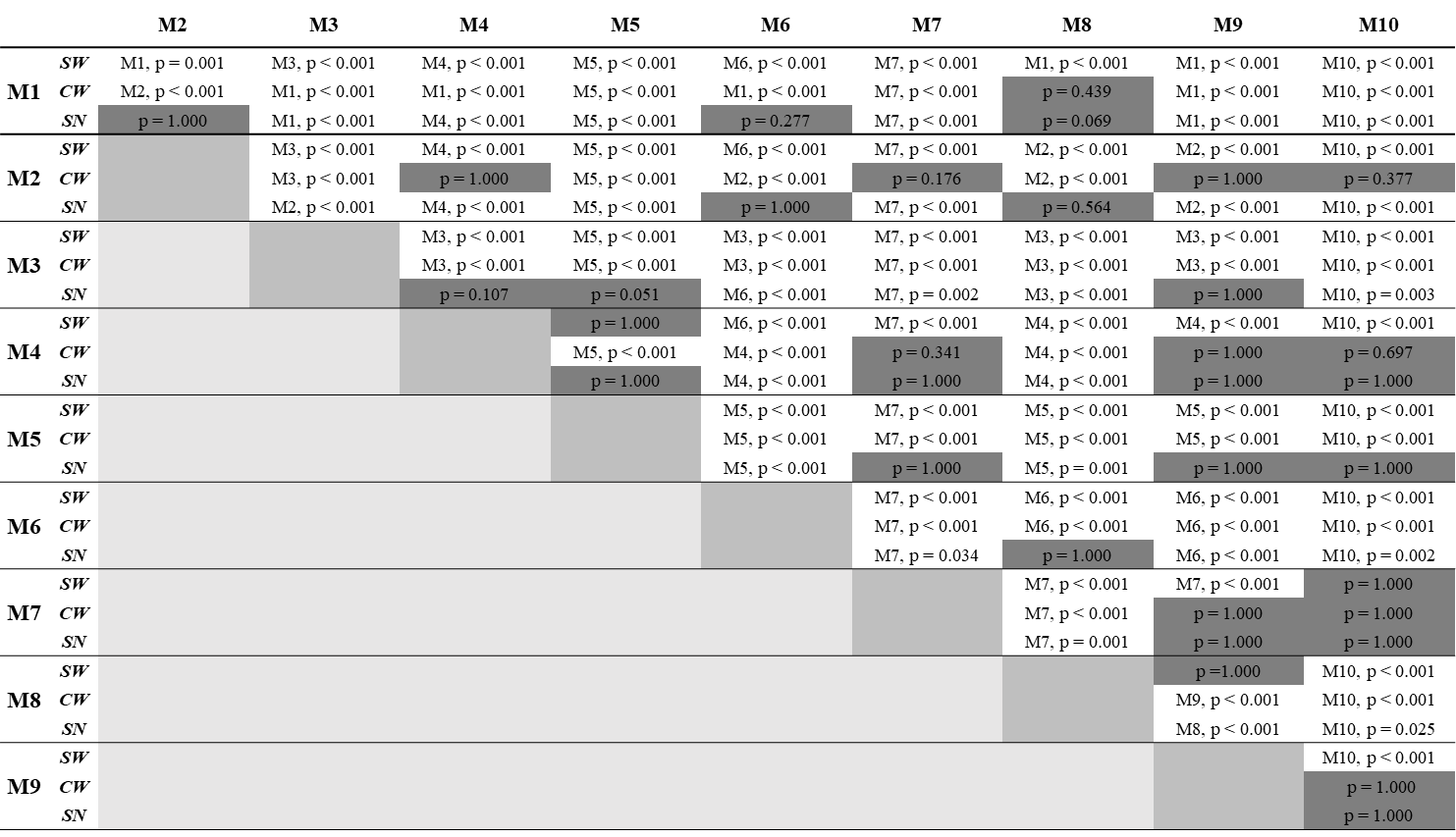


## Propagation of gait events inaccuacies on stride-level parameters

**Supplementary Table 3.** The correctly detected strides [%] for each method and walking conditions (straight-line walking, curvilinear walking and a step negotiation) are reported. The errors in the relevant stride duration [ms], length [mm], speed [mm/s], and stance/swing durations [ms] are described in terms of median (ME, *i.e.*, bias), inter-quartile range (IQRE, *i.e.*, precision), and median absolute errors (MAE, *i.e.*, accuracy); the relative errors [%] are also shown.

|  |  |  |  | **Stride Duration Error** | | | | | |  | **Stride Length Error** | | | | | |  | **Stride Speed Error** | | | | | |  | **Stance Duration Error** | | | | | |  | **Swing Duration Error** | | | | | |
| --- | --- | --- | --- | --- | --- | --- | --- | --- | --- | --- | --- | --- | --- | --- | --- | --- | --- | --- | --- | --- | --- | --- | --- | --- | --- | --- | --- | --- | --- | --- | --- | --- | --- | --- | --- | --- | --- |
|  |  |  |  | **ME** | | **IQRE** | | **MAE** | |  | **ME** | | **IQRE** | | **MAE** | |  | **ME** | | **IQRE** | | **MAE** | |  | **ME** | | **IQRE** | | **MAE** | |  | **ME** | | **IQRE** | | **MAE** | |
| **Strides [%]** | | |  | [$ms$] | [%] | [$ms$] | [%] | [$ms$] | [%] |  | [$mm$] | [%] | [$mm$] | [%] | [$mm$] | [%] |  | [$\frac{mm}{s}$] | [%] | [$\frac{mm}{s}$] | [%] | [$\frac{mm}{s}$] | [%] |  | [$ms$] | [%] | [$ms$] | [%] | [$ms$] | [%] |  | [$ms$] | [%] | [$ms$] | [%] | [$ms$] | [%] |
| **Straight-line Walking** | **M1** | 98.2 |  | 0 | 0.0 | 20 | 1.8 | 10 | 0.9 |  | 0.2 | 0.0 | 14.8 | 1.2 | 7.3 | 0.6 |  | 1.4 | 0.1 | 11.0 | 1.0 | 5.1 | 0.5 |  | -20 | -2.7 | 40 | 5.0 | 30 | 3.3 |  | 20 | 4.9 | 40 | 8.6 | 30 | 6.5 |
|  | **M2** | 93.0 |  | 0 | 0.0 | 30 | 2.2 | 10 | 1.1 |  | 0.8 | 0.1 | 27.8 | 2.1 | 13.7 | 1.0 |  | 1.1 | 0.1 | 12.4 | 1.2 | 6.1 | 0.6 |  | -60 | -7.9 | 90 | 8.8 | 60 | 8.3 |  | 60 | 14.6 | 80 | 16.6 | 60 | 15.0 |
|  | **M3** | 96.5 |  | 0 | 0.0 | 20 | 1.7 | 10 | 0.9 |  | -0.7 | -0.1 | 9.3 | 0.7 | 4.8 | 0.4 |  | 0.5 | 0.0 | 10.1 | 1.0 | 5.2 | 0.5 |  | 60 | 7.4 | 40 | 4.9 | 60 | 7.6 |  | -60 | -15.0 | 40 | 11.2 | 60 | 15.2 |
|  | **M4** | 98.8 |  | 0 | 0.0 | 30 | 2.4 | 10 | 1.0 |  | -0.3 | 0.0 | 13.0 | 1.0 | 6.6 | 0.5 |  | 0.3 | 0.0 | 13.6 | 1.3 | 6.6 | 0.6 |  | 50 | 6.0 | 50 | 8.1 | 50 | 6.3 |  | -50 | -12.2 | 50 | 15.0 | 50 | 12.5 |
|  | **M5** | 98.4 |  | 0 | 0.0 | 20 | 1.8 | 10 | 0.9 |  | -1.0 | -0.1 | 10.3 | 0.8 | 5.7 | 0.4 |  | 0.6 | 0.1 | 9.7 | 0.9 | 4.8 | 0.4 |  | 0 | 0.0 | 40 | 5.1 | 20 | 2.6 |  | 0 | 0.0 | 40 | 9.3 | 20 | 4.7 |
|  | **M6** | 98.8 |  | 0 | 0.0 | 30 | 2.4 | 10 | 1.0 |  | -0.5 | 0.0 | 16.3 | 1.2 | 8.3 | 0.6 |  | 1.0 | 0.1 | 11.1 | 1.0 | 5.2 | 0.5 |  | 10 | 1.2 | 40 | 5.5 | 20 | 2.8 |  | -10 | -2.6 | 40 | 10.7 | 20 | 5.1 |
|  | **M7** | 98.4 |  | 0 | 0.0 | 30 | 2.0 | 10 | 0.9 |  | -0.7 | -0.1 | 10.3 | 0.8 | 5.8 | 0.4 |  | 0.6 | 0.1 | 9.5 | 0.9 | 4.5 | 0.4 |  | -10 | -1.0 | 30 | 4.2 | 20 | 2.4 |  | 0 | 0.0 | 30 | 7.7 | 20 | 4.2 |
|  | **M8** | 94.6 |  | 0 | 0.0 | 30 | 2.2 | 10 | 0.9 |  | -0.2 | 0.0 | 20.5 | 1.5 | 9.9 | 0.7 |  | 0.0 | 0.0 | 15.0 | 1.4 | 7.6 | 0.7 |  | -10 | -1.4 | 120 | 17.7 | 40 | 5.1 |  | 10 | 2.2 | 110 | 31.7 | 40 | 8.8 |
|  | **M9** | 62.5 |  | 0 | 0.0 | 20 | 1.8 | 10 | 0.9 |  | 3.8 | 0.3 | 24.4 | 1.8 | 12.3 | 1.0 |  | 2.1 | 0.2 | 17.2 | 1.6 | 8.0 | 0.7 |  | -60 | -7.1 | 50 | 5.2 | 60 | 7.2 |  | 60 | 13.6 | 50 | 8.4 | 60 | 14.0 |
|  | **M10** | 98.5 |  | 0 | 0.0 | 30 | 2.0 | 10 | 0.9 |  | -0.8 | -0.1 | 10.3 | 0.8 | 5.8 | 0.5 |  | 0.6 | 0.1 | 9.6 | 0.9 | 4.5 | 0.4 |  | -10 | -1.1 | 40 | 4.3 | 20 | 2.4 |  | 0 | 0.0 | 30 | 7.4 | 20 | 4.3 |
|  |  |  |  |  |  |  |  |  |  |  |  |  |  |  |  |  |  |  |  |  |  |  |  |  |  |  |  |  |  |  |  |  |  |  |  |  |  |
| **Curvilinear Walking** | **M1** | 97.6 |  | 0 | 0.0 | 20 | 1.9 | 10 | 1.0 |  | 2.7 | 0.2 | 20.0 | 1.7 | 10.2 | 0.8 |  | 1.3 | 0.1 | 14.0 | 1.4 | 7.0 | 0.7 |  | -10 | -1.3 | 40 | 5.2 | 20 | 2.7 |  | 10 | 2.3 | 40 | 9.8 | 20 | 5.4 |
|  | **M2** | 88.9 |  | 0 | 0.0 | 90 | 7.7 | 40 | 3.9 |  | 0.6 | 0.0 | 47.9 | 3.8 | 23.5 | 1.9 |  | 0.5 | 0.0 | 45.5 | 4.5 | 22.4 | 2.2 |  | -40 | -6.0 | 110 | 14.2 | 70 | 9.1 |  | 50 | 11.8 | 108 | 26.8 | 70 | 17.5 |
|  | **M3** | 89.4 |  | 0 | 0.0 | 20 | 1.8 | 10 | 0.9 |  | -0.6 | 0.0 | 11.6 | 1.0 | 5.8 | 0.5 |  | -0.7 | -0.1 | 13.3 | 1.3 | 6.7 | 0.7 |  | 60 | 8.0 | 40 | 5.0 | 60 | 8.1 |  | -60 | -15.8 | 40 | 10.8 | 60 | 15.9 |
|  | **M4** | 69.9 |  | 10 | 0.8 | 80 | 6.6 | 40 | 3.3 |  | 3.3 | 0.3 | 44.2 | 3.6 | 20.9 | 1.8 |  | 0.0 | 0.0 | 32.6 | 3.0 | 16.3 | 1.5 |  | 30 | 3.9 | 90 | 12.3 | 50 | 7.2 |  | -20 | -5.1 | 90 | 24.5 | 50 | 13.2 |
|  | **M5** | 98.5 |  | 0 | 0.0 | 20 | 1.8 | 10 | 0.9 |  | 0.0 | 0.0 | 11.5 | 1.0 | 5.7 | 0.5 |  | -0.1 | 0.0 | 13.3 | 1.3 | 6.7 | 0.6 |  | 10 | 1.2 | 40 | 4.9 | 20 | 2.6 |  | -10 | -2.3 | 40 | 9.5 | 20 | 4.9 |
|  | **M6** | 77.3 |  | 10 | 0.8 | 80 | 6.8 | 40 | 3.3 |  | 6.5 | 0.5 | 65.6 | 5.5 | 30.3 | 2.6 |  | 4.0 | 0.3 | 37.6 | 3.7 | 19.0 | 1.8 |  | -10 | -1.3 | 90 | 12.3 | 40 | 5.5 |  | 10 | 2.7 | 90 | 21.9 | 40 | 10.5 |
|  | **M7** | 98.5 |  | 0 | 0.0 | 20 | 1.8 | 10 | 0.9 |  | 0.2 | 0.0 | 12.6 | 1.0 | 6.3 | 0.5 |  | 0.0 | 0.0 | 12.4 | 1.2 | 6.2 | 0.6 |  | 0 | 0.0 | 40 | 4.9 | 20 | 2.5 |  | 0 | 0.0 | 40 | 9.6 | 20 | 4.8 |
|  | **M8** | 97.8 |  | 0 | 0.0 | 70 | 5.8 | 30 | 2.9 |  | 1.0 | 0.1 | 214.2 | 18.9 | 95.9 | 8.5 |  | 3.0 | 0.3 | 139.4 | 14.6 | 64.3 | 6.5 |  | -80 | -11.5 | 150 | 20.1 | 90 | 12.6 |  | 90 | 25.0 | 150 | 39.0 | 90 | 25.6 |
|  | **M9** | 56.8 |  | 0 | 0.0 | 60 | 4.7 | 30 | 2.4 |  | 2.8 | 0.2 | 63.8 | 5.3 | 34.5 | 2.8 |  | 2.3 | 0.2 | 37.0 | 3.4 | 18.3 | 1.7 |  | -40 | -5.6 | 70 | 9.5 | 50 | 7.3 |  | 40 | 10.0 | 70 | 17.1 | 50 | 13.2 |
|  | **M10** | 98.4 |  | 0 | 0.0 | 20 | 1.8 | 10 | 0.9 |  | 0.2 | 0.0 | 12.6 | 1.0 | 6.3 | 0.5 |  | 0.0 | 0.0 | 12.3 | 1.2 | 6.1 | 0.6 |  | 0 | 0.0 | 40 | 5.0 | 20 | 2.5 |  | 0 | 0.0 | 40 | 9.8 | 20 | 4.8 |
|  |  |  |  |  |  |  |  |  |  |  |  |  |  |  |  |  |  |  |  |  |  |  |  |  |  |  |  |  |  |  |  |  |  |  |  |  |  |
| **Step Negotiation** | **M1** | 100.0 |  | -10 | -0.7 | 60 | 5.1 | 30 | 2.5 |  | 6.4 | 0.4 | 26.1 | 2.0 | 14.7 | 1.2 |  | 9.0 | 0.1 | 35.5 | 1.4 | 17.7 | 0.7 |  | -10 | -1.1 | 43 | 5.2 | 20 | 2.5 |  | 0 | 0.0 | 55 | 13.5 | 30 | 7.3 |
|  | **M2** | 87.6 |  | 0 | 0.0 | 90 | 6.6 | 40 | 3.3 |  | 12.8 | 1.1 | 66.3 | 5.4 | 33.2 | 2.6 |  | 7.8 | 0.0 | 39.3 | 4.5 | 20.3 | 2.2 |  | -60 | -7.1 | 75 | 8.2 | 70 | 7.7 |  | 60 | 15.3 | 70 | 16.8 | 65 | 16.1 |
|  | **M3** | 7.9 |  | -30 | -2.3 | 58 | 4.6 | 30 | 2.8 |  | 2.5 | 0.2 | 27.6 | 1.8 | 15.1 | 0.9 |  | 14.3 | -0.1 | 38.3 | 1.3 | 21.3 | 0.7 |  | 10 | 1.7 | 100 | 14.1 | 40 | 4.9 |  | -50 | -10.6 | 63 | 16.9 | 50 | 10.6 |
|  | **M4** | 96.6 |  | -10 | -0.8 | 50 | 4.0 | 25 | 2.0 |  | 4.6 | 0.4 | 27.7 | 2.3 | 15.3 | 1.3 |  | 5.0 | 0.0 | 25.6 | 3.0 | 17.3 | 1.5 |  | 50 | 6.5 | 50 | 5.9 | 50 | 6.9 |  | -50 | -13.7 | 50 | 13.9 | 50 | 13.7 |
|  | **M5** | 100.0 |  | -10 | -0.7 | 50 | 3.7 | 20 | 1.8 |  | 5.3 | 0.5 | 24.4 | 1.9 | 11.4 | 0.9 |  | 9.0 | 0.0 | 28.4 | 1.3 | 16.2 | 0.6 |  | 0 | 0.0 | 20 | 2.9 | 10 | 1.4 |  | -10 | -2.4 | 58 | 13.6 | 30 | 7.0 |
|  | **M6** | 100.0 |  | -10 | -0.7 | 63 | 5.5 | 30 | 2.7 |  | 8.2 | 0.6 | 32.6 | 2.5 | 17.0 | 1.4 |  | 5.7 | 0.3 | 33.6 | 3.7 | 19.4 | 1.8 |  | 20 | 2.6 | 50 | 6.2 | 30 | 3.9 |  | -20 | -5.0 | 60 | 14.3 | 30 | 7.3 |
|  | **M7** | 100.0 |  | -10 | -0.7 | 43 | 3.9 | 20 | 1.9 |  | 6.4 | 0.5 | 23.4 | 1.9 | 13.1 | 1.0 |  | 7.8 | 0.0 | 28.7 | 1.2 | 17.0 | 0.6 |  | 0 | 0.0 | 40 | 4.9 | 20 | 2.4 |  | -10 | -2.5 | 60 | 15.1 | 30 | 7.8 |
|  | **M8** | 95.5 |  | -10 | -0.7 | 168 | 13.5 | 80 | 6.5 |  | 4.0 | 0.4 | 419.3 | 37.4 | 203.3 | 18.0 |  | 4.2 | 0.3 | 224.5 | 14.6 | 113.4 | 6.5 |  | 5 | 0.6 | 145 | 18.5 | 50 | 6.2 |  | 0 | 0.0 | 100 | 27.3 | 35 | 9.4 |
|  | **M9** | 18.0 |  | -15 | -1.0 | 55 | 4.3 | 30 | 2.2 |  | 16.6 | 1.4 | 60.0 | 4.7 | 29.8 | 2.4 |  | 11.9 | 0.2 | 31.6 | 3.4 | 20.5 | 1.7 |  | -30 | -3.8 | 58 | 7.4 | 40 | 5.5 |  | 30 | 8.6 | 58 | 13.8 | 50 | 11.1 |
|  | **M10** | 100.0 |  | -10 | -0.7 | 40 | 3.7 | 20 | 1.9 |  | 6.6 | 0.5 | 23.5 | 1.9 | 13.0 | 1.0 |  | 7.8 | 0.0 | 28.3 | 1.2 | 16.4 | 0.6 |  | 0 | 0.0 | 40 | 4.9 | 20 | 2.4 |  | -10 | -2.4 | 50 | 13.0 | 30 | 7.1 |
